# Personality traits as risk factors for relapse or recurrence in major depression: a systematic review

**DOI:** 10.3389/fpsyt.2023.1176355

**Published:** 2023-05-05

**Authors:** Nada Altaweel, Rachel Upthegrove, Andrew Surtees, Buse Durdurak, Steven Marwaha

**Affiliations:** ^1^School of Psychology, Institute for Mental Health, University of Birmingham, Birmingham, United Kingdom; ^2^Department of Psychology, Princess Nourah Bint Abdulrahman University, Riyadh, Saudi Arabia; ^3^Birmingham Woman's and Children's NHS Foundation Trust, Birmingham, United Kingdom; ^4^Specialist Mood Disorders Clinic, Birmingham and Solihull Mental Health NHS Foundation Trust, Birmingham, United Kingdom

**Keywords:** personality traits, relapse, recurrence, depression, personality disorders

## Abstract

**Background:**

Major depressive disorder (MDD) is highly recurrent. Identifying risk factors for relapse in depression is essential to improve prevention plans and therapeutic outcomes. Personality traits and personality disorders are widely considered to impact outcomes in MDD. We aimed to evaluate the role of personality aspects in the risk of relapse and recurrence in MDD.

**Method:**

A PROSPERO-registered systematic review was conducted using Medline, Embase, PsycINFO, Web of Science and CINAHL as data sources, together with hand searching of four journals over the five years till 2022. There was independent abstract selection, quality assessment and data extraction from each study.

**Results:**

Twenty two studies me t eligibility criteria involving 12,393 participants. Neurotic personality features are significantly associated with the risk of relapse and recurrence of depression, though the data is not uniform. There is some, though limited, evidence that borderline, obsessive-compulsive and dependent personality traits or disorders increase the risk for relapse in depression.

**Limitations:**

The small number, in addition to the methodological heterogeneity of the included studies, did not allow further analysis, such as meta-analysis.

**Conclusion:**

People with high neuroticism and dependent personality traits, borderline personality disorder or obsessive-compulsive personality disorder, compared to those without, may be at a higher risk of experiencing relapse or recurrence of MDD. Specific and targeted interventions may potentially reduce relapse and recurrence rates in these groups and could improve outcomes.

**Systematic review registration:**

https://www.crd.york.ac.uk/prospero/display_record.php?RecordID=235919, identifier: CRD42021235919.

## 1. Introduction

Depression currently affects around 25.8 million people worldwide (1) and is a major public health challenge (2). It is a highly recurrent disorder (3), adding to its illness burden. A return of the symptoms of MDD can be described by the term “*relapse*,” originally adapted from the work of Frank et al. (4), where they provided an operational criterion based on consistent, empirical evidence for each term that represents a response of the course of illness of depression and has been widely used by researchers in the field. They defined *relapse* as a reappearance of depressive symptoms after a partial remission but prior to full recovery, where the term *recurrence* was defined as the re-emergence of an episode of depression after achieving full recovery (4). The return of depressive symptoms is common, with 50% of people who have had one depressive episode and recovered likely to have one or more episodes during their lifetime, and around 80% of people who have had more than two episodes at a high risk of experiencing additional depressive episodes within 5 years (5).

One way to address the ongoing burden of major depression is to identify the risk factors for relapse and recurrence. Most of the available research in this domain has focused on clinical aspects as risk factors of depressive relapse; for example, evidence from a systematic review found an association between depressive relapse and the severity level of symptoms, residual symptoms, and the number of previous episodes (6). Other studies have addressed this issue by investigating and comparing different types of interventions as preventative for relapse in depression, such as antidepressants, mindfulness, and CBT (7, 8).

Several studies in the literature have linked personality with different outcomes of depression. Personality traits can be defined as “enduring patterns of perception, relation and thinking of the environment and oneself that are expressed in a wide variety of social and personal contexts” (9). The DSM system classifies personality disorders using a categorical approach. On the other hand, The International Classification of Diseases 11th Revision (ICD-11) lists five domains of personality traits as descriptions of personality pathology, which is an official adoption of the dimensional model of personality (10). Therefore, researchers tend to refer to these two international systems when investigating issues regarding personality disorders and depression. However, the differences in the approach taken to the study, clinical coding and understanding of how to investigate personality traits that cause difficulties to people show that there is a far from settled position amongst clinicians and researchers alike.

Evidence in the literature has been seen in terms of the connection between mood disorders, including depression, and personality disorders and that having comorbidity between them can lead to worse long-term outcomes. For example, Tyrer et al. (11) investigated the long-term outcome in patients with mixed symptoms of depression, anxiety, general neurotic syndrome, and ICD-11 personality disorders. Patients with one or a mix of these disorders (*n* = 210) were recruited to a randomized controlled trial receiving different treatments for ten weeks (medication, placebo, CBT, and self-help) and then were followed up for 30 years. Findings showed that patients with mood and personality disorders had a worse outcome compared to those with one mood disorder and no personality disorder.

Personality traits could also be a potentially important factor in understanding depressive relapse. For instance, neuroticism and negative emotionality are associated with new-onset depression in children and adolescents; the parameters may overlap. Still, a major part of the literature is based on “depressive personality,” which raises methodological issues about the definition and cause and effect (12).

Although efforts have been made to clarify the relationship between personality traits and the risk of relapse and recurrence in depression, available findings about this connection are inconsistent. Buckman et al. (6) highlighted in their systematic review the risk factors for relapse and recurrence of depression. They focused on neuroticism as a personality factor which appeared in their review to be associated with the risk of recurrence in depression, yet other personality factors did not seem to be presented in that review. Another systematic review reported that evidence on some personality traits and relapse or recurrence of depression lacked replicated results (3). Therefore, highlighting personality traits related to relapse and recurrence of depression could contribute to synthesizing the available evidence, enhance understanding of this phenomenon and provide a comprehensive perception of it.

This systematic review aims to investigate what personality traits are associated with the relapse or recurrence of major depression in adults.

## 2. Methods

### 2.1. Study protocol

The systematic review protocol was registered in the International Prospective Register of Systematic Reviews (PROSPERO) in February 2021 (protocol ID: CRD 42021235919); https://www.crd.york.ac.uk/prospero/display_record.php?RecordID=235919.

### 2.2. Eligibility criteria

The inclusion criteria were: [a] population of adult participants aged 18 years and older who had experienced at least one previous episode of Major Depressive disorder (MDD) and were diagnosed according to an internationally recognized diagnostic system (the Diagnostic and Statistical Manual of Mental Disorders DSM or International Statistical Classification of Diseases ICD) using clinical interview and/ or validated depression measures; [b] studies investigated relapse or recurrence defined by a clinical interview; [c] studies must also investigate at least one personality trait, where authors use standardized personality instruments; published in the English language. The exclusion criteria were: [a] MDD is not the primary diagnosis (e.g., anxiety, psychosis, substance misuse); [b] studies are (*N* = 1) design (case reports), cross-sectional studies, or no follow-up period allowing the outcome to occur.

### 2.3. Search strategy

Five databases were searched: Medline, CINAHL, Embase, Web of Science and Psychinfo. The searches were carried out in January 2023 without date restrictions. In addition, four relevant journals were searched from 2018 to 2022 (i.e., *Clinical Psychology Review, Journal of Consulting and Clinical Psychology, Depression Research and Treatment*, and *Depression and Anxiety*). The search strategy involved both key terms and subject heading techniques and MeSH headings where appropriate. Search terms included **1#** Personality traits OR Personality Types OR Personality characteristics OR Emotional dysregulation OR Emotional regulation deficits OR affective instability OR impulsivity OR Mood instability AND **2#** Depression OR Major Depressive Disorder OR Major depression OR MDD AND **3#** Risk factors OR Predictors OR association AND **4#** Relapse OR depressive relapse OR worsening OR recurrent OR recurrence. The search strategy for each search engine is available in a Supplementary material.

### 2.4. Selection of articles

All citations were downloaded into EndNote, and duplicates were removed. Titles and abstracts of selected studies in the first phase search were screened by two independent authors (NA, BD) in light of eligibility criteria; then, eligible studies were retrieved in full. Inter-rater reliability was assessed. Any disagreement was discussed and resolved by consensus. Two reviewers extracted data independently. Data extracted from identified papers were study author, date, country, population, participant characteristics, study features, clinical data, and results (personality traits associated with relapse or recurrence in people with MDD), including statistical tests used and measures of effect size where available.

### 2.5. Quality assessment

Eligible studies were assessed for the risk of bias using the Newcastle-Ottawa Scale (NOS) (13) by two independent authors (NA, BD). It is a scale designed to assess the quality of non-randomized studies, such as case-control and cohort studies. Each study is judged using a star system on three broad aspects: selection, comparability of the groups, and the outcome (13).

### 2.6. Data extraction and synthesis

Two reviewers (NA, BD) extracted data independently, and all included studies were included in a narrative synthesis.

### 2.7. Outcome

The main outcome was relapse *or* recurrence of MDD among adults, diagnosed through a clinical assessment. This approach was taken as confusion remains with regard to distinguishing between depression relapse and recurrence; therefore, the current review assessed studies that investigated the association between personality traits and relapse or recurrence.

## 3. Results

### 3.1. Study selection

**Figure 1:**
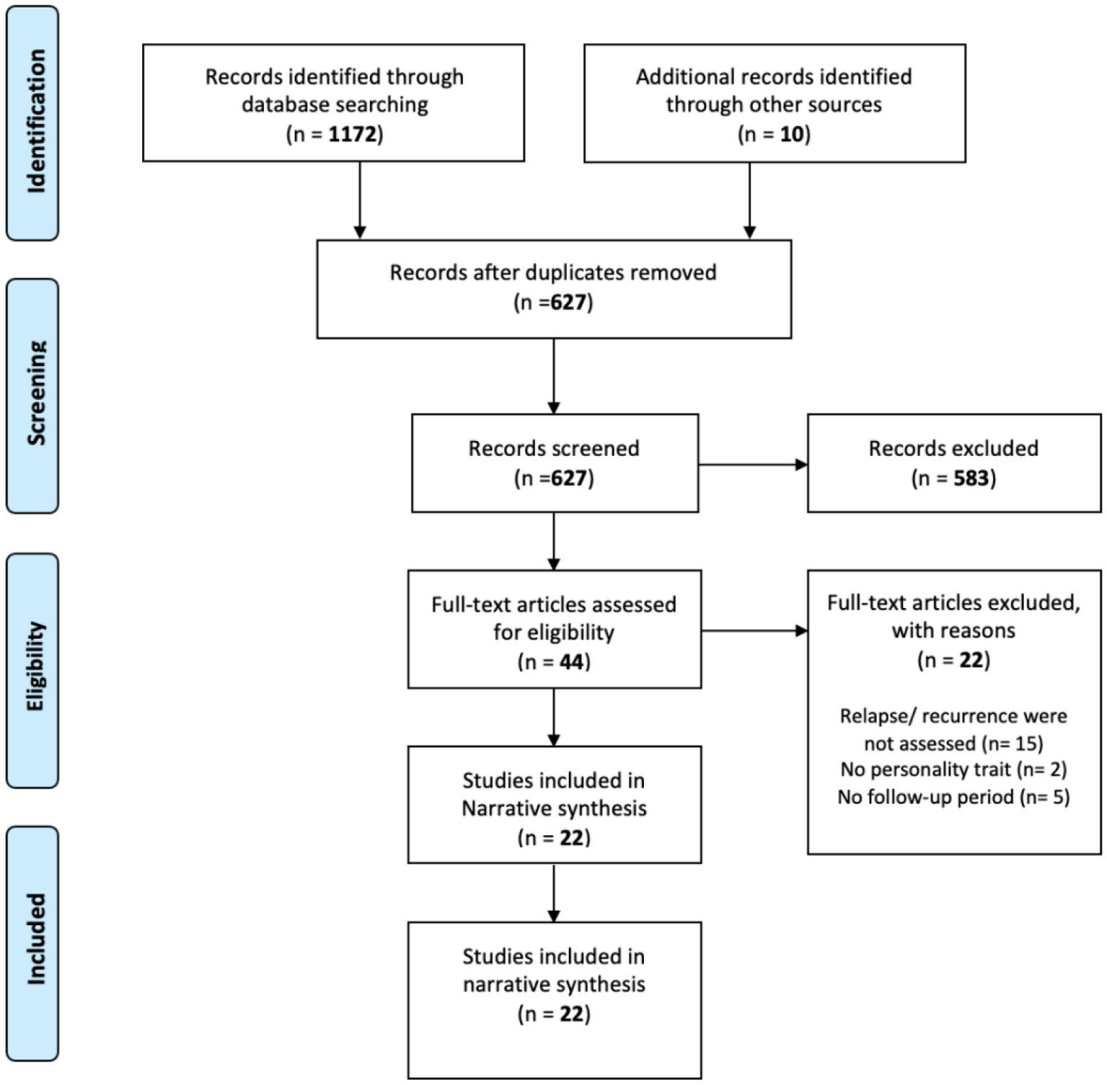
Flow-diagram of the study selection process.

The search process retrieved 1182 studies (Web of Science *n* = 467, PsycINFO *n* = 375, Embase *n* = 189, Medline *n* = 85, CINAHL *n* = 56, and additional resources *n* = 10). Figure 1 shows a PRISMA (2009) flow diagram of the study selection process. De-duplication resulted in 627 remaining studies, which underwent abstract and title screening. A total of 44 studies were retrieved for full-text review. The inter-rater reliability for the full-text screening was generally moderate (kappa = 81.25%), and 22 studies were eligible according to the current review criteria.

### 3.2. Study characteristics

The current review included 22 prospective studies that were published in the English language. Studies addressed personality traits as risk factors for relapse (*n* = 9), recurrence (*n* = 11) or both (*n* = 2) in MDD. Most studies were conducted in the USA (*n* = 6) or the Netherlands (*n* = 6). Two studies were conducted in Finland, and the other individual studies were from the UK, New Zealand, Norway, Canada, Mexico, Spain, Denmark, and Japan. The follow-up period of these studies varied between 6 months and 13 years. Details of the characteristics of the included studies are shown in Table 1. Most studies did not use the personality traits as described in the DSM-5 or the ICD-10/11, instead using well personality schedules such as the big-5 factor model of personality.

**Table 1:** Characteristics of the included studies.

**Study**	**Country**	**Study of relapse or recurrence**	**Personality traits**	**Instruments**	**Sample**	**Mean age**	**Gender**	**Follow-up period**
Mulder et al. (14)	New Zealand	Both	Novelty seeking, Harm avoidance, Reward dependence, Self-directedness, Cooperativeness, Self-transcendence, and Axis II personality disorders	(SCID-II), and the temperament and character inventory	*N* = 175	31.6 years	57% female	6 months
de Klerk- Ssuis et al. (15)	The Netherlands	Relapse	Self- compassion	The self compassion scale [SCS; (16)]	*N*= 282	50.3	67.7 female	15 months
Melartin et al. (17)	Finland	Recurrence	Neuroticism	The eysenck personality inventory: dimension of neuroticism	*N =* 269	41 years	72% female	18 months
O'Leary and Costello (18)	UK	Relapse	Axis II personality disorders, Extroversion and Neuroticism	Personality assessment was based on the informant rated standard assessment of personality [SAP, (19)] and the self-rated Maudsley Personality Inventory [MPI, (20)]	*N* = 84	39 years	58% female	18 months
Berlanga et al. (21)	Mexico	Recurrence	Neuroticism, Extroversion, Psychoticism, Desire for social acceptance	Eysenck personality questionnaire	*N* = 42	36 years	76% female	1 year
Segal et al. (22)	Canada	Relapse	Dependency, Self-Criticism	Dysfunctional attitude scale DAS	*N =* 59	38.43 years	40.6% female	1 year
Gopinath et al. (23)	US	Relapse	Neuroticism, Self- efficacy	the NEO Personality Inventory Neuroticism Scale and the self-efficacy scale for managing depression	*N =* 386	45.7 years	74.65% female	1 year
Gollan et al. (24)	US	Relapse	Avoidant, Dependent, Passive Aggressive, Self-Defeating	MCMI II = Millon Clinical Multiaxial Inventory, Second Edition	*N =* 93	37.5 years	79.6% female	2 years
Hardeveld et al. (25)	The Netherlands	Recurrence	Neuroticism	The twelve-item subscale of the NEO Five-Factor Inventory (NEO-FFI) Questionnaire	*N =* 375	40.3 years	66.9% female	2 years
Noteboom et al. (26)	The Netherlands	Recurrence	Neuroticism, extraversion, openness to experience, agreeableness and conscientiousness	The Dutch 60-item self-report NEO five-factor inventory (NEO-FFI)	*N =* 1085	42.4 years	64.9% female	2 years
Steunenberg et al. (27)	The Netherlands	Recurrence	Neuroticism, extraversion, openness to experience, agreeableness, and conscientiousness	The NEO five factor Inventory	*N* = 92	76	66% female	3 years
Skodol et al. (28)	US	Recurrence	Axis II personality disorders	SCID-II	*N =* 1996	30 to over 50	67.5% female	3 years
Verhoeven et al. (29)	The Netherlands	Both	Neuroticism, Extraversion, Openness to experience, Agreeableness, Conscientiousness, Mastery, Loneliness, self-esteem	The five-factor inventory (NEO-FFI), the mastery Scale, the Rosenberg self-esteem scale and the Loneliness scale	*N =* 213	43.1 years	64.8% female	3 years
Asano et al. (30)	Japan	Recurrence	Harm Avoidance, Self-Directedness	the Temperament and Character Inventory (TCI)	*N =* 109	55.3 years	61.46% female	3–11 years
Spinhoven et al. (15)	The Netherlands	Relapse	Neuroticism, Experiential avoidance	the Dutch version of the 60-item NEO five-factor inventory, the Dutch version of the 9-item acceptance and action questionnaire-I	*N =* 2513	44.1 years	Mean 553.3 female	4 years
Ilardi et al. (31)	US	Relapse	Axis II Personality disorders	The personality disorder examination [PDE; (32)]	*N =* 50	38.3 years	78% female	M = 49.9 months
Holma et al. (33)	Finland	Recurrence	Axis II Personality disorders, Neuroticism and Extroversion	(SCID- II) The eysenck personality inventory	*N =* 163	42.3 years	73% female	5 years
Bukh et al. (34)	Denmark	Recurrence	Axis II Personality disorders, Neuroticism and Extroversion	(SCID- II) The Eysenck Personality Inventory	*N* = 301	36 years	66.1% female	5 years
Grilo et al. (35)	US	Relapse	Schizotypal, borderline, avoidant, and obsessive- compulsive personality disorders	The diagnostic interview for DSM-IV personality disorders (DIPD-IV; 29)	*N =* 303	33.3 years	65% female	6 years
Alnaes and Torgersen (36)	Norway	Relapse	Axis II Personality disorders, Self-doubt, Insecurity, Sensitivity, Dependency Compliance, Emotional instability, Rigidity, Severe superego, Parsimony, Indecision, Orderliness Exhibitionism, Imagination, Sociability, Aggression, Emotional expressiveness	Structured interview for DSM-III personality disorders (SIDP-I) Millon clinical multiaxial inventory (MCMI-I) Basic character inventory (BCI)	*N =* 298	35 years	69% female	6 years
Serrano et al. (37)	Spain	Recurrence	Agreeableness, conscientiousness, extraversion, neuroticism and openness to experience	The Big Five inventory (BFI-10)	*N* = 3102	61.65 years	53.91% female	6.9 years
Bromberger et al. (38)	US	Recurrence	Trait anxiety, Private self- consciousness, Dispositional optimism	The 10-item modified version of the State-Trait Personality Inventory (39, 40). The 10-item Self-Consciousness Scale – Revised (41). The 6-item Life Orientation Test (41).	*N* = 443	45 years	100% female	13 years

DSM axis II personality disorders are avoidant -borderline – impulsive- anankastic -dependent - paranoid -histrionic, and schizoid. SCID is the structured clinical interview for DSM-III-R personality disorders.

### 3.3. Participants

The eligible studies recruited a total of 12,393 participants, including both cases and healthy controls, with a mean age of approximately 41.5 years, and females represented about 67% of the total participants in these studies; see Table 1 for details. Participants were assessed for the symptoms of MDD using measures that included the Hamilton rating scale for depression (HAM-D), Beck Depression Inventory, Structured Clinical Interview for DSM-III-R—Patient Version (SCID-P) and The Composite International Diagnostic Interview (CIDI), Lifetime Version 2.1 (WHO Lifetime Version 2.1).

### 3.4. Quality assessment of the included studies

Using the Newcastle-Ottawa scale, the risk of bias scores for identified studies were generally classified as high-quality studies in which four studies scored 7 (*n* = 4), nine studies scored 8 (*n* = 9), and nine studies scored 9 out of 9 (*n* = 9); see Table 2 for details.

**Table 2:** Quality assessments scores of the included studies.

**Study**	**Selection**	**Comparability**	**Outcome**	**Total score**
Steunenberg et al. (27)	^***^	^**^	^**^	7
Spinhoven et al. (42)	^****^	^**^	^*^	7
Grilo et al. (35)	^****^	^*^	^**^	7
Serrano et al. (37)	^***^	^**^	^**^	7
O'Leary and Costello (18)	^****^	^*^	^***^	8
Alnaes and Torgersen (36)	^****^	^*^	^***^	8
Segal et al. (22)	^****^	^**^	^**^	8
Gopinath et al. (23)	^***^	^**^	^***^	8
Gollan et al. (24)	^****^	^**^	^**^	8
Hardeveld et al. (25)	^***^	^**^	^***^	8
Verhoeven et al. (29)	^****^	^**^	^**^	8
de Klerk-Sluis et al. (15)	^***^	^**^	^***^	8
Bromberger et al. (38)	^***^	^**^	^***^	8
Noteboom et al. (26)	^****^	^**^	^***^	9
Skodol et al. (28)	^****^	^**^	^***^	9
Asano et al. (30)	^****^	^**^	^***^	9
Ilardi et al. (31)	^****^	^**^	^***^	9
Holma et al. (33)	^****^	^**^	^***^	9
Mulder et al. (14)	^****^	^**^	^***^	9
Melartin et al. (17)	^****^	^**^	^***^	9
Berlanga et al. (21)	^****^	^**^	^***^	9
Bukh et al. (34)	^****^	^**^	^***^	9

Newcastle-Ottawa scale for assessment of the quality of included studies—Cohort studies (each asterisk represents if individual criterion within the subsection was fulfilled).

### 3.5. Personality traits and the risk of relapse/recurrence in MDD

**Figure 2:**
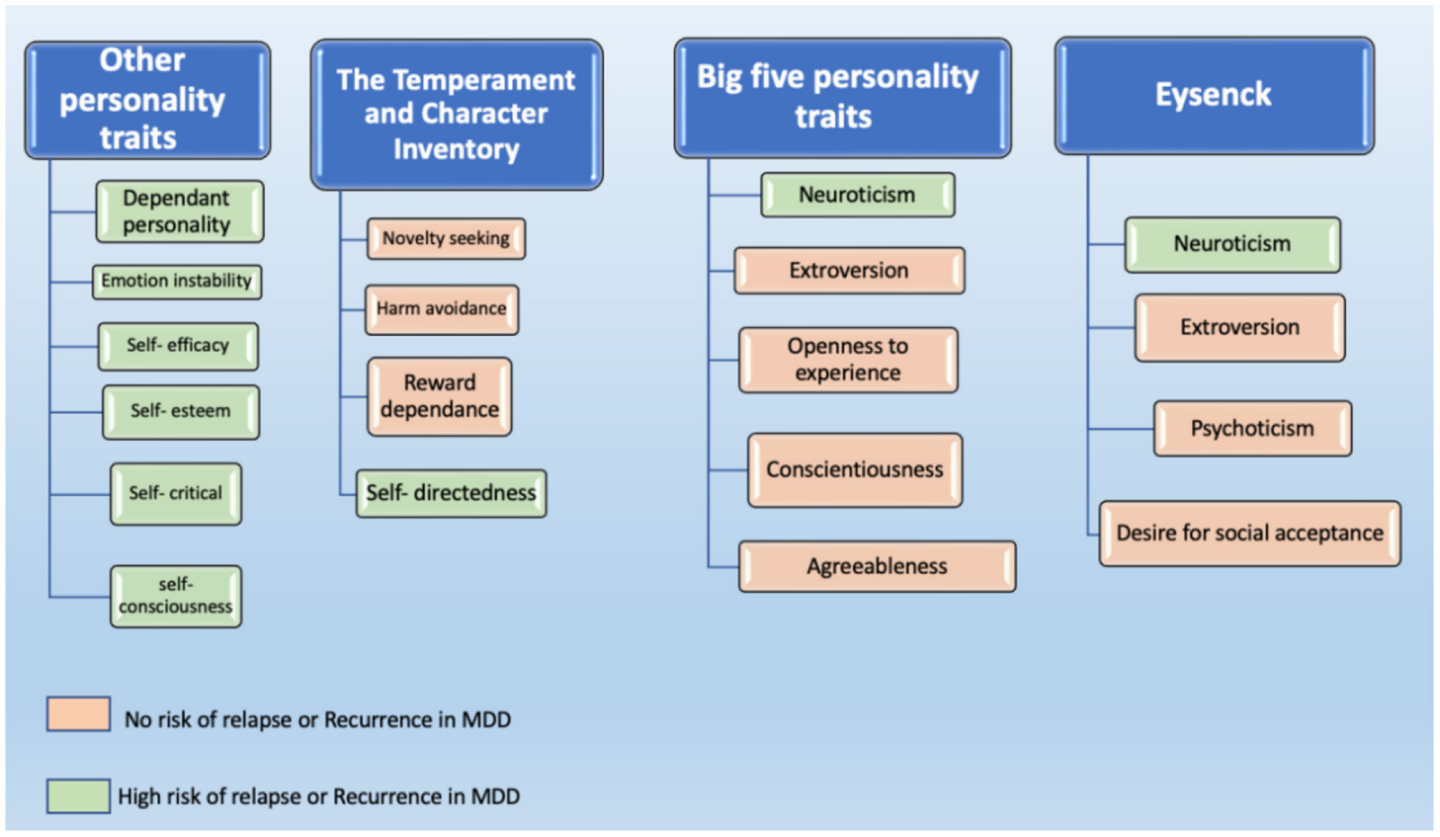
Personality traits that have been reported to be associated with the risk of relapse or recurrence in MDD.

In summary, existing studies have investigated personality traits in four broad ways. These are personality traits that fall under the Eysenck Personality Inventory, the Big Five Personality Traits Inventory (NEO), the Temperament and Character Inventory (TCI), and other types of personality traits/assessments (see Figure 2).

#### 3.5.1. Eysenck personality inventory

Six studies used the self-rated Maudsley Personality Inventory [MPI, (20)] or the Eysenck Personality Inventory with regard to depression relapse/recurrence. The traits include neuroticism, extroversion, psychoticism, and the desire for social acceptance. Two studies reported a relationship between an elevated score of neuroticism and an increased risk of relapse or recurrence (17, 21). Furthermore, two studies assessed the subscale of both neuroticism and extraversion concerning depression relapse/recurrence. A higher than median score on the neuroticism and extraversion subscales did not predict depression relapse (18). These findings are consistent with six-year and five-year follow-up studies that found no significant relationship between neuroticism or extraversion and recurrence in depression (33, 34).

#### 3.5.2. Big five personality traits

Eight studies have investigated the relationship between relapse or recurrence and some personality traits of the NEO Five-Factor Inventory, which comprise neuroticism, extraversion, openness to experience, conscientiousness, and agreeableness. The findings of these studies varied; only two studies reported that neuroticism did not predict depression relapse (42) or recurrence (25); however, generalizability issues might arise from these findings, as one study dealt with a sample of patients in specialized mental health care where patients are more likely to have severe, chronic symptoms compared to those in primary health care (25). Therefore, the sample is unlikely to be representative of the entire population of depression patients. Additionally, residual depressive symptoms that have been proven to be an important risk factor for depression were not included in the analysis, which may limit the robustness of the findings. The other study reported issues regarding the attrition of the sample during the follow-up period, which may have limited the generalizability of the results in that study also (42).

In contrast, six studies revealed a significant association between neuroticism and relapse/recurrence. The first reported that a high score on the neuroticism scale was significantly related to relapse of depressive disorder. Still, this association did not remain after a multi-variate regression (23). Two other studies found that neuroticism was the only one of the Big Five personality traits that showed a significant association with recurrence in both univariate and multivariate analyses (26, 27).

Furthermore, a study has classified patients with depression according to their different relapse patterns into four groups: “quick symptom decline,” “slow symptom decline,” “steady residual symptoms,” and “high residual symptoms.” They found that patients with depression who relapsed with increasing depressive symptoms showed a high score on the neuroticism subscale and a low score on the extraversion subscale compared to other patients who relapsed with different patterns (i.e., slow decline, quick decline, and steady residual depressive symptoms) (29).

Additionally, a study of 3102 participants who were followed for around six years by Serrano et al. (37) investigated gender differences in some depression outcomes, including recurrence, and the association between these outcomes and personality traits of the big five inventory. The study revealed that higher neuroticism was associated with MDD recurrence in women, whereas agreeableness was associated with reduced MDD recurrence only in men. Finally, the study showed that there was a significant association between conscientiousness and the recurrence of depression symptomology only in women, while openness increased the risk of recurrence in men (37).

#### 3.5.3. The temperament and character inventory

Two studies have investigated the link between relapse/recurrence in major depression and personality structures assessed using the TCI, including novelty seeking, harm avoidance, reward dependence, self-directedness, cooperativeness, and self-transcendence. The first reported no significant relationship between the personality traits of TCI and depressive relapse (14). In contrast, a four-year prospective follow-up study found that patients with low scores on self-directedness (SD) showed significantly shorter times to recurrence compared to patients with high SD scores. At the same time, harm avoidance (HA) did not predict recurrent depression (30).

#### 3.5.4. Other groups of personality traits/assessments

Several researchers have assessed several personality traits using other types of personality assessments to determine the role of personality traits in the risk of relapse and recurrence of major depression.

A prospective study assessing the contribution of four personality patterns (i.e., Avoidant, Dependent, Passive Aggressive, and Self-Defeating) to the risk of depressive relapse found that only dependent personality was associated with a high risk of relapse in depression (24). This is consistent with another study that revealed that among 17 different personality traits assessed using the Basic Character Inventory (BCI), dependency and emotional instability were the only significant predictors of relapse in major depression (36). Further, a study with a cohort of 386 primary care patients suggested that relapse was significantly associated with lower scores on the self-efficacy scale for managing depression (23). In addition, lower self-esteem scores also appeared to be associated with the risk of relapse (29), while self-critical individuals emerged as more prone to relapse, especially in the case of experiencing adverse life events related to achievement (22). Furthermore, de Klerk-Sluis et al. (15) did not find a significant association between self-compassion and relapse in 282 remitted depression patients. Finally, a 13-year follow-up study used the 10-item self-consciousness Scale – Revised (41) to assess the trait of private self-consciousness and its relation to recurrent MDD. The study revealed that for every 1-point increase in the scale score, a tendency toward self- or internal-focused attention increased the risk of recurrent MDD by 6% (38).

### 3.6. Personality disorders and the risk of relapse and recurrence in MDD

Eight of the 22 studies assessed the contribution of personality disorders that fall under the ICD-10 or the DSM IV Axis II personality disorders (avoidant, borderline, impulsive, anankastic, dependent, paranoid, histrionic, and schizoid) to the risk of relapse or recurrence in MDD. There were some conflicting findings of these studies, in which four did not find any significant association between the DSM IV Axis II personality disorders and relapse (14, 18) nor recurrence (28, 33). At the same time, in their six-year follow-up with over 300 participants, Grilo et al. (35) found that MDD patients with personality disorders, particularly borderline personality disorder BPD and obsessive-compulsive personality disorder OCPD, had a significantly shorter time to relapse compared to MDD patients without personality disorders. These findings are consistent with the results of the six-year follow-up study of Alnaes and Torgersen (36), where they found that BPD predicted relapse. Moreover, DSM Axis II personality disorders have been observed to be a significant risk factor for depression relapse in a study by Ilardi et al. (31), though they did not identify which personality disorder was related to relapse. Finally, Bukh et al. (34) reported an increase in recurrence rate by 80% in patients with comorbid cluster C personality disorders (Avoidant, dependent, and obsessive-compulsive personality disorders).

## 4. Discussion

This systematic review aimed to investigate the contribution of personality traits to the risk of relapse and recurrence in MDD. In summary, the majority of studies investigating neuroticism suggest it is associated with an increased risk of relapse or recurrence in major depression. Additionally, borderline personality disorder, obsessive-compulsive personality disorder, and dependent personality style all appeared to be linked with relapse. Other factors which could be considered to be personality traits, such as self-criticism, lower self-esteem, lower self-efficacy, lower self-directedness, and self-consciousness, also appear to be important. Emotional instability is associated with relapse in depression, though it is also a feature of borderline personality disorder.

Several interpretations of the connection between personality traits and depression have been provided in the literature. With regard to neuroticism, a potential explanation indicated by some researchers is that there are factors that mediate the association between neuroticism and depression. For example, studies have suggested that rumination on sadness is a possible mediator between depressive symptoms and neuroticism (43, 44). Furthermore, another study revealed that cognitive reactivity, particularly suicidal thinking and hopelessness, have been found to mediate the association between neuroticism and depression, where depression patients with such thinking patterns are prone to the recurrence of depression (45). Finally, previous evidence indicates that personality features such as neuroticism and conscientiousness have a genetic association with major depression, and this may also partly explain our findings (46).

Dependent personality is also important in explaining relapse and recurrence in MDD. There is a considerable amount of evidence linking dependency with the development of mood disorders, specifically depression (47). According to the DSM-5, the core feature of dependent personality disorder “is the extreme need to be taken care of which leads to submissive behavior and fears of separation” (9). The interpretation presented in the psychological literature for this link is that dependent persons tend to respond to situations such as rejection, separation, or personal loss with feelings of hopelessness and helplessness, which are also features of MDD. In other words, the interaction between stressful events and the characteristics of dependent individuals makes them vulnerable to depression (47).

Personality disorders, particularly BPD and OCPD, appeared to be associated with depressive relapse/recurrence in the current review. On a cognitive level, one study found that “functional impairment and erroneous interpretations of intrusive thoughts” in OCPD patients predicted depressive symptoms (48). This functional impairment could mediate the relationship between OCPD and MDD. Likewise, the co-morbidity between BPD and MDD has been widely recognized in the literature (49–51), which may explain the relapse phenomenon among depressed patients with BPD. To illustrate, common characteristics have been found between BPD and MDD; for example, one study revealed that participants with high BPD/MDD showed greater emotional dysregulation and difficulties in controlling impulsive behavior compared to other participants with low BPD/MDD symptoms (52).

Our findings are in keeping with previous literature, in smaller, less representative samples, that has demonstrated the potential for comorbid personality disorders and poor treatment response in depression. A meta-analysis by Newton-Howes et al. (53) found that depressed patients with comorbid personality disorders are twice likely to have a poorer outcome than patients with only depression. Neglecting personality disorders' role in worsening treatment outcomes in depression can lead to several issues, such as overprescription of medication (54) and the planning of ineffective interventions (55). Therefore, it is important to emphasize examining factors like personality in customizing interventions for depression (56).

The present review showed that several personality traits had not received sufficient research attention, although signals exist on a possible relationship between these traits and depressive relapse. For instance, emotional regulation is a factor that might be associated with relapse and recurrence in MDD. In a review by Compare et al. (57), clinical studies demonstrated that emotional regulation is a significant element in developing MDD. Furthermore, emotional dysregulation is prospectively associated with incident depression over 18 months (58). Similarly, there appeared to be no relevant studies investigating the impact of irritability on depressive relapse.

One interpretation of these findings related to emotional dysregulation is that depression is associated with an impairment in cognitive control, such as processing unpleasant components. This is linked with higher rumination, expressive suppression and impaired cognitive reappraisal, which are significant aspects of emotional dysregulation (57). Another systematic review found that individuals who recovered from depression reported higher maladaptive emotional regulation strategies than healthy participants who had never experienced depression. These studies, whilst limited in range and number, suggest emotional dysregulation deficits may be important in the genesis, manifestation (59) and outcomes of depression, such as relapse (60).

Impulsivity is another trait that could be linked to depressive relapse, yet it has received little clinical attention. A meta-analytical review stated that a strong relationship has been found between impulsivity and remitted depression, which continues even in remission (61); all included studies in that review reported high impulsivity scores among participants with MDD. However, it has been observed that many studies that addressed the association between impulsivity and depression have done so in light of suicidal behavior, so it remains unclear how far impulsivity may impact depression when suicidality is not a feature. In addition, although existing attempts to investigate the association between impulsivity and relapse depression, there is no sufficient explanation as to the critical aspects of impulsivity. Therefore, it may be essential to explore specific aspects of impulsivity (e.g., non-planning, cognitive impulsivity and impulsive decision-making) and their influence on relapse or recurrence in depression.

### 4.1. Limitations of the literature

The available studies that addressed personality disorders in terms of their relationship with relapse and recurrence in MDD were not recent, the last one being published in 2016. This resulted in relying on standard classification systems such as the DSM and the ICD-10, which have since been further developed. For example, the classification of personality disorders in the ICD-11 has changed significantly from the ICD-10. The ICD-11 takes a dimensional approach and emphasizes severity and functional impact (10, 62). Likewise, the DSM-5 has adopted the maladaptive personality traits and the level of personality functioning as key features of personality pathology (63, 64), but it remains a categorical diagnosis in that system.

Similarly, most available studies did not use the personality frameworks used in DSM or ICD and assessed personality using a number of other frameworks and with differing instruments. The differences in classification systems, their development, as well as the lack of uniformity in how personality is conceptualized across scientific studies in the field mean that results are difficult to compare. Newton-Howes et al. (65) examined the difference between three approaches to personality disorders taxonomy (dichotomous, dimensional, and severity) in depressed patients (*n* = 578); to assess which of these approaches has the most significant clinical utility in terms of predicting symptoms reduction. The outcomes of interest were psychopathology and social functioning. To achieve this, the study analyzed data from four clinical trials at six weeks to six-month outcomes; that used different interventions for depression. The results revealed that all three approaches had contributed significantly to assessing personality disorders at the six-month outcome, and no specific taxonomy exceeded the other two.

Whilst studies investigated the association between personality and relapse or recurrence, we cannot, of course, infer causation from them. In addition, it can be said that research findings on the area of relapse and personality traits are generally inconsistent. This might be due to the relatively limited number of studies or how personality factors are conceived and measured (12). For instance, personality trait/disorder measures were administered at different time points in these studies, which could have affected the results. Previous research demonstrated that the number of personality disorders decreased in the case of assessment during recovery compared to the evaluation during the course of illness with both self-report measures and structured interviews (66). Additionally, the variation could be due to the lack of control of some critical clinical variables reported in the literature to be strongly associated with depressive relapse, such as the number of previous episodes and their severity (6).

### 4.2. Limitations of this review

A small number of studies were included in this review, which was a significant limitation. The small number, in addition to the heterogeneity in the methods of these studies, precluded further analysis, such as meta-analysis. The hand-searching process resulted in additional potential eligible articles that were not found through searching the databases, which is a limitation concerning the search strategy. However, this could be due to the inaccurate indexing of some articles in databases or the applied search limits, such as restricting the search to English. On the other hand, several strengths can be considered, such as the pre-registration of the study protocol, searches using multiple electronic databases alongside hand-searching, and the use of a quality assessment tool that indicated the high quality of all included studies. Furthermore, the consistency of our findings with previous studies validates our conclusions. Finally, the present review contributes to the area of personality and relapse in depression, which, to our knowledge, is the largest study of its kind.

### 4.3. Future directions

A limited number of studies have explored the long-term outcome of depression in relation to personality dysfunction, though it is widely clinically assumed that they are strongly related. Future studies need to examine personality factors which may be transdiagnostic (e.g., emotional dysregulation) (67) and that are already linked to aspects of depression (68, 69) but which are under-investigated in terms of the links with relapse and recurrence. Ideally, cohort studies should examine the impacts of personality traits on incidence, recovery, persistence and relapse to provide a deep level understanding of this area.

In addition, an interpretation of the mechanisms in which personality traits interact or lead to recurrent depression is needed, as this area remains ambiguous, as well as other factors that may overlap personality aspects (i.e., stressful events). Finally, how far the influence of personality factors changes with age is also unknown, though it could aid in investigating the clinical staging of mood disorders.

## 5. Conclusions

Given the morbidity and mortality associated with relapse in MDD, the literature regarding personality factors and relapse and recurrence is relatively scant. However, there does appear to be a signal for neuroticism, dependent, obsessional and borderline personality features being important. In addition, researchers should attempt to address some personality traits common in a range of mood disorders, such as emotional dysregulation and impulsivity, which have, in fact, rarely been the subject of investigations concerning their effect on recurrence or relapse. Understanding the risks for relapse or recurrence of depression is essential, as it could significantly improve therapeutic and prevention plans; this, in turn, would reflect a significant development in the mental health field.

## Data availability statement

The original contributions presented in the study are included in the article/Supplementary material, further inquiries can be directed to the corresponding author.

## Author contributions

NA, SM, RU, and AS conceived the presented idea. NA and BD carried out the systematic literature search, extracted the data, and assessed the quality of the included studies. NA wrote the manuscript with input from all authors. All authors discussed the results and provided critical feedback.
