# Supplementary material for: Personality traits as risk factors for relapse or recurrence in major depression: a systematic review
Source: Front Psychiatry. 2023 May 5;14:1176355. doi: 10.3389/fpsyt.2023.1176355 (PMC10196019; doi:10.3389/fpsyt.2023.1176355)
Supplement: Supplementary file 1 [file Data_Sheet_1.DOCX]

Supplementary Material

Personality traits as risk factors for relapse or recurrence in major depression: a systematic review

Nada Altaweel^*^, Rachel Upthegrove, Andrew Surtees, Buse Durdurak, Steven Marwaha

*** Corresponding author:** Nada Altaweel: nxa981@ student.bham.ac.uk

# Search strategy

**Embase**

Map Term to Subject Heading**,** Limit: English language.

**#1** (Personality traits or Personality Types or Personality characteristics or Emotional dysregulation or Emotional regulation deficits, or affective instability or impulsivity or Mood instability).mp. [mp=title, abstract, heading word, drug trade name, original title, device manufacturer, drug manufacturer, device trade name, keyword heading word, floating subheading word, candidate term word].

**2#** (Depression or Major Depressive Disorder or Major depression or MDD).mp. [mp=title, abstract, heading word, drug trade name, original title, device manufacturer, drug manufacturer, device trade name, keyword heading word, floating subheading word, candidate term word].

**3#** (Risk factors or Predictors or association).mp. [mp=title, abstract, heading word, drug trade name, original title, device manufacturer, drug manufacturer, device trade name, keyword heading word, floating subheading word, candidate term word].

**4#** (Relapse or depressive relapse or worsening or recurrence or recurrent).mp. [mp=title, abstract, heading word, drug trade name, original title, device manufacturer, drug manufacturer, device trade name, keyword heading word, floating subheading word, candidate term word].

**5#** 1 AND 2 AND 3 AND 4

**Medline**

Map Term to Subject Heading**,** Limit: English language.

**1#** (Personality traits or Personality Types or Personality characteristics or Emotional dysregulation or Emotional regulation deficits, or affective instability or impulsivity or Mood instability).mp. [mp=title, abstract, original title, name of substance word, subject heading word, floating sub-heading word, keyword heading word, organism supplementary concept word, protocol supplementary concept word, rare disease supplementary concept word, unique identifier, synonyms].

**2#** (Depression or Major Depressive Disorder or Major depression or MDD).mp. [mp=title, abstract, original title, name of substance word, subject heading word, floating sub-heading word, keyword heading word, organism supplementary concept word, protocol supplementary concept word, rare disease supplementary concept word, unique identifier, synonyms].

**3#** (Risk factors or Predictors or association).mp. [mp=title, abstract, original title, name of substance word, subject heading word, floating sub-heading word, keyword heading word, organism supplementary concept word, protocol supplementary concept word, rare disease supplementary concept word, unique identifier, synonyms].

**4#** (Relapse or depressive relapse or worsening or recurrence or recurrent).mp. [mp=title, abstract, original title, name of substance word, subject heading word, floating sub-heading word, keyword heading word, organism supplementary concept word, protocol supplementary concept word, rare disease supplementary concept word, unique identifier, synonyms].

**5#** 1 AND 2 AND 3 AND 4

**APA PsychINFO**

Map Term to Subject Heading**,** Limit: English language.

**1#** (Personality traits or Personality Types or Personality characteristics or Emotional dysregulation or Emotional regulation deficits, or affective instability or impulsivity or Mood instability).mp. [mp=title, abstract, heading word, table of contents, key concepts, original title, tests & measures, mesh].

**2#** (Depression or Major Depressive Disorder or Major depression or MDD).mp. [mp=title, abstract, heading word, table of contents, key concepts, original title, tests & measures, mesh].

**3#** (Risk factors or Predictors or association).mp. [mp=title, abstract, heading word, table of contents, key concepts, original title, tests & measures, mesh].

**4#** (Relapse or depressive relapse or worsening or recurrence or recurrent).mp. [mp=title, abstract, heading word, table of contents, key concepts, original title, tests & measures, mesh].

**5#** 1 AND 2 AND 3 AND 4

**Web of Science**

ALL= (Personality traits OR Personality Types OR Personality characteristics OR Emotional dysregulation OR Emotional regulation deficits, OR affective instability OR impulsivity OR Mood instability) AND ALL= (Depression OR Major Depressive Disorder OR Major depression OR MDD) AND ALL= (Risk factors OR Predictors OR association) AND ALL= (Relapse OR depressive relapse OR worsening OR recurrence OR recurrent)**.**

**CINAHL**

"Personality traits OR Personality Types OR Personality characteristics OR Emotional dysregulation OR Emotional regulation deficits, OR affective instability OR impulsivity OR Mood instability" OR (MH "Multiple-Personality Disorder") OR (MH "Emotional Regulation") OR (MH "Avoidant Personality Disorder") OR (MH "Dependent Personality Disorder") OR (MH "Passive-Aggressive Personality Disorder") OR (MH "Histrionic Personality Disorder") OR (MH "Antisocial Personality Disorder") AND (MH "Depression") OR "Depression OR Major Depressive Disorder OR Major depression OR MDD" AND (MH "Risk Factors") OR "Risk factors OR Predictors OR association" AND (MH "Recurrence") OR "Relapse OR depressive relapse OR worsening OR recurrence OR recurrent".

**
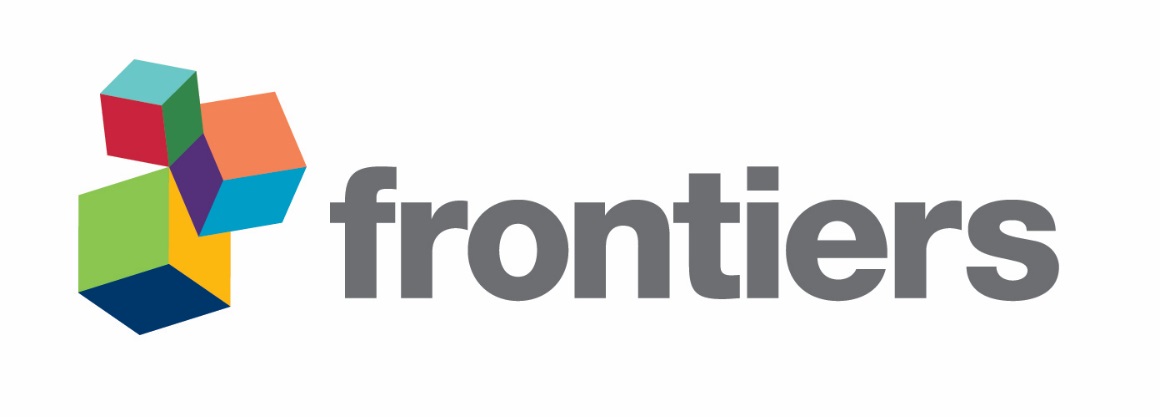
**
